# Altered olfactory responses in *Fmr1* KO mice

**DOI:** 10.1038/s41598-024-80000-5

**Published:** 2025-01-23

**Authors:** Jan Tuma, Amtul-Noor Rana, Teena Philip, Jeong Ben Park, Hye Young Lee

**Affiliations:** 1 The Department of Cellular and Integrative Physiology, The University of Texas Health Science Center at San Antonio, San Antonio, TX USA; 2 Department of Pathophysiology, Faculty of Medicine in Pilsen, Charles University, Alej Svobody 1655/76, 323 00 Plzen, Czech Republic

**Keywords:** Olfactory bulb, Olfactory behavior, Odor discrimination test, Fragile X syndrome, *Fmr1* KO mice, Olfactory system, Developmental biology, Neuroscience

## Abstract

Fragile X syndrome (FXS) is a neurodevelopmental disorder oftentimes associated with abnormal social behaviors and altered sensory responsiveness. It is hypothesized that the inappropriate filtering of sensory stimuli, including olfaction, can lead to aberrant social behavior in FXS. However, previous studies investigating olfaction in animal models of FXS have shown inconsistent results. Here, we found that *Fmr1* knock-out (KO) mice, a mouse model of FXS, showed increased sniffing duration for non-social odors during their first exposure. Additionally, while wild-type (WT) males demonstrated differences in behavioral patterns between non-social odors while *Fmr1* KO males did not show such distinction. We also showed that *Fmr1* KO males spent significantly less time sniffing female urine odor compared to WT males. Moreover, we found an increased volume of the olfactory bulb in *Fmr1* KO males. Overall, our findings suggest that the *Fmr1* KO mice demonstrate atypical olfactory behaviors as well as structural changes in the olfactory bulb.

**Supplementary Information:**

The online version contains supplementary material available at 10.1038/s41598-024-80000-5.

## Introduction

Fragile X syndrome (FXS) is one of the most common forms of inherited intellectual disabilities and the most frequent monogenic cause of autism spectrum disorders (ASDs)^1^. It arises from a loss-of-function mutation in the fragile X messenger ribonucleoprotein 1 (*FMR1*) gene that encodes the fragile X messenger ribonucleoprotein (FMRP), an mRNA-binding protein^2^. FXS manifests various symptoms including learning disabilities, alterations in social behavior, arousal impairments, and heightened sensitivity to sensory stimuli^3–5^. Some abnormal social behavior patterns may be influenced by inappropriate filtering of sensory stimuli and consequently altered sensory responsiveness across various modalities^5–9^. Understanding sensory processing might help advance our knowledge on the link between behavioral responsiveness and the neurobiology of FXS and ASDs. Of particular interest is the role of smell perception as it is capable of eliciting potent emotional responses that profoundly influence social and cognitive development^10,11^. Social chemosignaling is also an important sensory component that mediates human social interactions^12,13^. Previous studies have shown that altered olfaction and smell perception might be the mechanistic link between the abnormal smell sensory response and impaired social communication associated with ASDs^14,15^. Although there are very few studies investigating sensory response in FXS patients alone, Rogers et al. (2003) demonstrated that children with either FXS or ASDs exhibited abnormal olfactory sensitivity, without showing significant differences between FXS and ASD groups^16^. Furthermore, anatomical and structural changes related to olfactory abnormalities in FXS patients have not been extensively studied. However, empirical neuroimaging and bioinformatic data from ASD patients suggest that olfactory symptoms could be related to abnormalities in the olfactory bulb and/or prefrontal cortex^17,18^. Therefore, it is critical to investigate the anatomy and behaviors governed by the olfactory system to understand social behavioral changes in FXS.

Animal models of FXS have also shown abnormal sensory responsiveness in auditory^19–21^, visual^22^, and olfactory processing^6^, mirroring symptoms observed in FXS and ASD patients. Specifically, reduced olfactory attraction and aversion has been observed in the *Drosophila* model of FXS (*dfmr1*^*−*^)^23^. This deficit arises from impaired neuronal inhibitory connections in the antennal lobe. Such compromised olfactory encoding consequently leads to impaired olfactory behaviors in *dfmr1*^*−*^ flies and it is hypothesized that this mechanism might be ubiquitously present in the brain of FXS patients^23^. Studies in mouse models have also found deficits in olfactory sensing, however, the behavioral nature of this impairment has continued to be controversial. For instance, Larson et al. (2008) used a two-alternative olfactory reinforced responding paradigm and reported normal olfactory sensitivity and decreased odor discrimination in *Fmr1* knock-out (KO) mice^24^. Conversely, Schilit Nitenson et al. (2015) conducted a spontaneous olfactory cross-habituation task and observed decreased olfactory sensitivity and normal discrimination behavior in *Fmr1* KO mice^25^. The observed discrepancies in results could arise from the difference in experimental methods. In fact, the two-alternative reinforced choice paradigm can be conceptualized as an experiment that is essentially opposite to that of a cross-habituation task and requires top-down learning processes with operant conditioning and the consolidation of a stimulus-reward association^6^.

Previous studies have shown that olfactory function can also correlate with the structure and anatomy of the olfactory bulb (OB)^26,27^. OB interneurons play a crucial role in odor detection, discrimination, and learning by modulating the neural activity of excitatory projection neurons^28^. Specifically, within its glomerular layer (GL), inhibitory neurons facilitate the relay of odorant information from olfactory sensory neuron axons to mitral/tufted cells^29^. This transmission is regulated by OB granule cells (GCs) in the granule cell layer (GCL), which are generated in the subventricular zone (SVZ) and migrate to the GL and GCL of OB^30^. Additionally, periglomerular GABAergic interneurons expressing Calbindin (CalB^+^), Calretinin (CalR^+^) and tyrosine hydroxylase (TH^+^) are three of the most commonly studied periglomerular interneuron subtypes, and play a key role in periglomerular layer olfactory processing within the OB^29^. The importance of postnatal and adult neurogenesis for the formation of these interneurons should be emphasized with respect to the role of FMRP in this process^31,32^*.* Interestingly, Castren et al. 2005 demonstrated that the mouse and human FMRP-deficient neurospheres generated more neurons (threefold and fivefold, respectively) than control neurospheres, and these cells showed morphological alterations, such as fewer and shorter axons and a smaller cell body volume^33^. Thus, we can hypothesize that the abnormal neurogeneration seen in *Fmr1* KO mice^31,32^ may be reflected not only in the function but also in the OB anatomical characteristics.

In this study, our aims are twofold: (1) identify and characterize altered olfactory behaviors to provide additional insights regarding social deficits associated with FXS, and (2) conduct morphological analyses of the OB in *Fmr1* KO mice. We demonstrate that *Fmr1* KO mice did not show significant difference in their ability to sense non-social and social odors compared to WT mice during habituation/dishabituation test. However, both genders of *Fmr1* KO mice showed greater interest when first exposed to a non-social odor. Additionally, *Fmr1* KO males had a decreased olfactory response after being exposed to female urine, but not to male urine. We further revealed that *Fmr1* KO males had a larger OB volume than WT males. Detailed analyses showed that the increased OB volume is not due to a disproportionate cell density in neither the GL nor the GCL. Together, our results indicate that the mouse model for FXS show different patterns of olfactory responses from WT mice when exposed to non-social odors and social odors, as well as show anatomical differences from WT mice.

## Materials and methods

### Materials

**Table:** 

Reagent or resource	Source	Identifier
***ANTIBODIES***		
Anti-NeuN mouse IgG1, clone A60, 1:500	Chemicon®	MAB377; RRID: AB_2314889
Anti-Calbindin chicken IgY, 1:500	Encor Biotechnology Inc	CPCA-Calb; RRID: AB_2572237
Anti-Calretinin chicken IgY, 1:500	Encor Biotechnology Inc	CPCA-Calret; RRID: AB_2572241
Anti-Tyrosine Hydroxylase chicken IgY, 1:500	Encor Biotechnology Inc	CPCA-TH; RRID:AB_2737416
Alexa Fluor® 488 AffiniPure Donkey Anti-Mouse IgG (H + L), 1:500	Jackson ImmunoResearch Laboratories, Inc	715–545-151; RRID:AB_2341099
Alexa Fluor® 647 AffiniPure Donkey Anti-Chicken IgY (H + L), 1:500	Jackson ImmunoResearch Laboratories, Inc	703–605-155; RRID:AB_2340379
***CHEMICALS***		
Parafromaldehyde (PFA), 96%, extra pure	ACROS Organics™	41678–0010
D(+)-Sucrose, 99.7%, for biochemistry	ACROS Organics™	AC177140010
Sodium Citrate Dihydrate	Fisher BioReagents	BP327-500
10X Phosphate Buffered Saline (PBS)	Fisher BioReagents	BP3994
Fluriso™, Isoflurane, USP	VetOne	502017
DAPI (4’,6-diamidin-2-fenylindole, dihydrochloride)	Invitrogen	D1306
ProLong Gold Antifade Reagent	Invitrogen	P36934
Goat Serum, New Zealand origin	Gibco	16210–064
Triton X-100	ThermoFisher Scientific	A16046.AP
McCormick Banana extract	McCormick & Company, Inc	N/A
McCormick Vanilla extract	McCormick & Company, Inc	N/A
Gelatin from porcine skin (Type A)	Sigma-Aldrich	G2500-500G
Cresyl Violet acetate	Sigma-Aldrich	C5042-10G
Wright-Giemsa Stain	Volu-Sol	VWG-032
Ethanol (EtOH)	DECON LABORATORIES, INC	64–17-5
Methanol (MeOH)	Fisher Chemical	A412-4
***MATERIALS***		
Cotton-Tipped Applicators STERILE	MCKESSON	MFR# 24–106-1S
Superfrost Plus Microscope Slides	ThermoFisher Scientific	12–550-15
Permount® Mounting Medium	Fisher Chemical	SP15-500
Gelatin-coated slides	FD NeuroTechnologies	PO101
Tissue-Plus O.C.T. Compound	Fisher Healthcare	23–730-571
Embedding Molds	ThermoSCIENTIFIC	2219
***EXPERIMENTAL MODELS: Organisms/strains***		
Mouse: FVB.129P2-*Pde6b*^+^ *Tyr*^*c-ch*^/AntJ	Jackson Laboratory	004828; RRID: IMSR_JAX:004828
Mouse: FVB.129P2-*Pde6b*^+^ *Tyr*^*c-ch*^* Fmr1*^*tm1*^^*Cgr*^/J	Jackson Laboratory	004624; RRID: IMSR_JAX:004624
***SOFTWARE and ALGORITHMS***		
Fiji (ImageJ2) v.2.0.0-rc-69/1.52p	National Institutes of Health, USA	Imagej.net
Zeiss ZEN 2.3 software	Carl Zeiss Microscopy GmbH	www.zeiss.com
R v.3.6.0	R-project.org	cran.r-project.org

### Methods

#### Animals

Wild-type (WT, stock #004828, FVB.129P2-*Pde6b*^+^ *Tyr*^*c-ch*^/AntJ), and *Fmr1* KO (stock #004624, FVB.129P2-*Pde6b*^+^ *Tyr*^*c-ch*^* Fmr1*^*tm1Cgr*^/J) mice were obtained from Jackson Laboratory. These *Fmr1* KO mice were originally designed using a neomycin resistance cassette targeted to exon 5 of the *Fmr1* gene^34^. All experiments were performed in 3 month-old male and female mice. Heterozygous females (*Fmr1*^+/-^) were generated by crossing *Fmr1* KO female (*Fmr1*^-/-^) and WT male (*Fmr1*^+/y^) mice, which were then crossed with WT male (*Fmr1*^+/y^) mice to obtain *Fmr1* KO males (*Fmr1*^-/y^) and littermate WT males (*Fmr1*^+/y^) as a control group. *Fmr1* KO females (*Fmr1*^-/-^) and WT females (*Fmr1*^+/+^, control group) were generated by crossing hemizygous male (*Fmr1*^-/y^) and homozygous female *Fmr1* KO (*Fmr1*^-/-^) or WT male (*Fmr1*^+/y^) and WT female (*Fmr1*^+/+^), respectively. Both groups were kept separately and matched by the day of birth. All mice were housed in the university’s animal facility, which was maintained at standard humidity and temperature conditions with a 12-h light–dark cycle. Food and water were provided ad libitum. All procedures were approved by the University of Texas Health Science Center at San Antonio (UTHSCSA) Institutional Animal Care and Use Committee (IACUC) and were conducted in accordance with guidelines in the Animal Welfare Regulations and the Guide for the Care and Use of Laboratory Animals within a fully AAALAC-accredited facility. The study is reported in accordance with ARRIVE guidelines^35^.

#### Olfactory behavioral assay

A modified olfactory habituation/dishabituation behavioral test^36,37^ was used to determine whether WT and *Fmr1* KO mice differ in their ability to distinguish between non-social odors or social odors, as well as habituate to an odor after continuous exposure. We exposed mice to non-social odors first in order to establish their olfactory function, and then the social odors were presented in order to evaluate their social response ability.

**Fig. 1:**
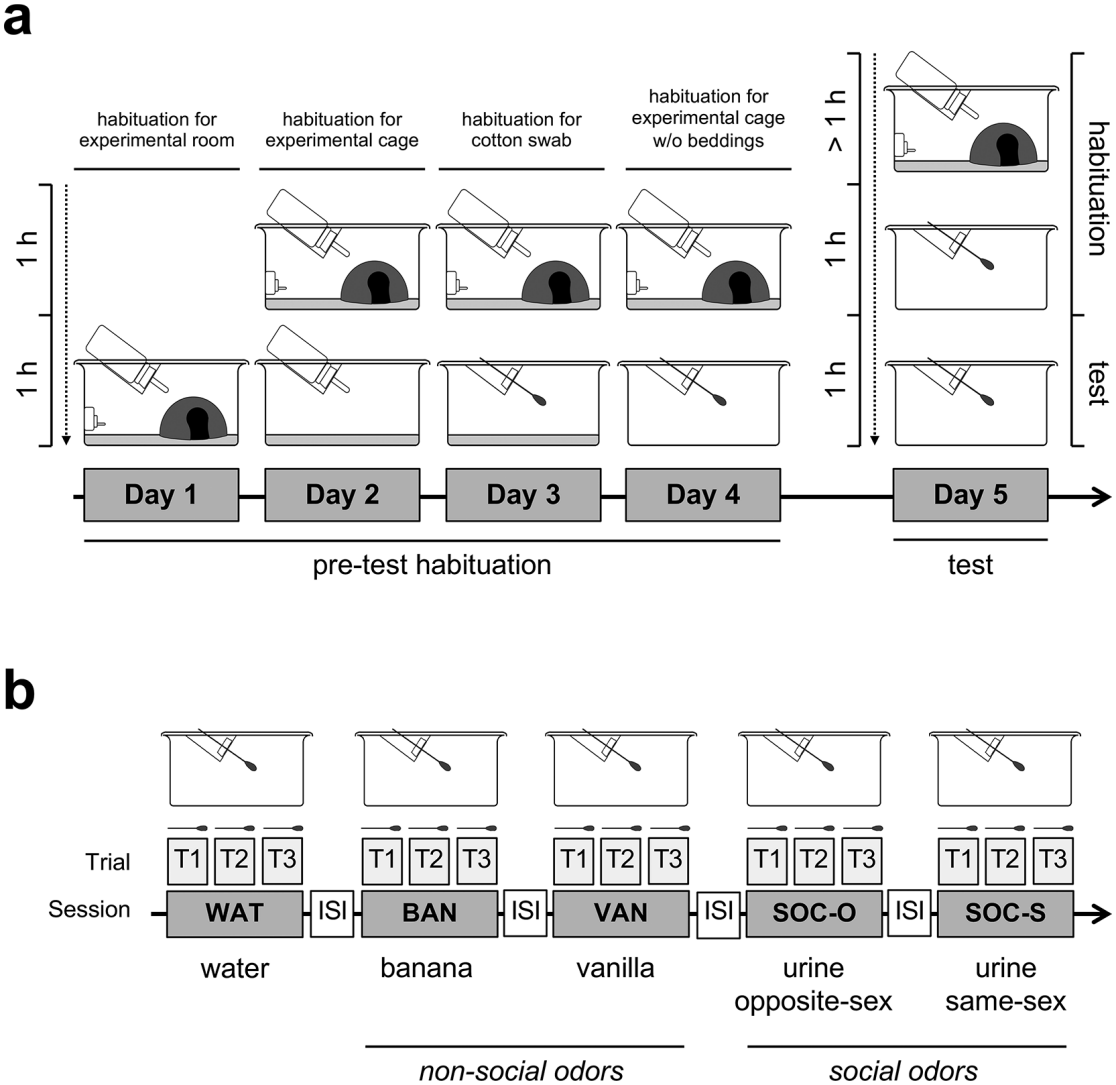
Scheme of the habituation for experimental conditions and description of olfactory habituation/dishabituation task.** (a)** Scheme of the experimental procedure. The protocol consisted of pre-test habituation for experimental condition (Day 1 – Day 4) and behavioral task (Day 5). Before the start of the behavioral assay, mice were also habituated for experimental room condition (at least 1 h) and empty experimental cage containing the clean cotton swab. **(b)** Scheme of the olfactory habituation/dishabituation task. Mice were presented with cotton swab soaked with same odorant three times (T1–T3). Different olfactory cues were used in following order: odorless water (WAT), serving as a baseline for mouse interest in the cotton swab, banana (BAN), vanilla (VAN), mouse urine from opposite sex (SOC-O) and mouse urine from same sex (SOC-S) then tested mouse sex-paired group. Each odor session was interrupted with 2 min inter-session interval (ISI).

*Pre-test habituation* Animals were habituated in the experimental room and testing conditions for four days (Fig. 1a). On the first day of habituation (Day 1), animals were transferred to the experimental room and kept in their home cages for 1 h, which contained shelter, an air nozzle for the air supply inlet, and a filled water bottle. The next day (Day 2), animals were kept in the experimental room in their home cage for 1 h and then single-caged into experimental cages for 1 h, which contained bedding, a filled water bottle, and food pellets. The experimental cages did not contain air nozzle or shelter, as these artifacts had shown to distract mice during the olfactory habituation/dishabituation assay. On Day 3, the animals were kept in the experimental room in their home cage for 1 h, and then single-caged into experimental cages for 1 h, which only contained bedding and an odorless cotton swab. On the last day of habituation (Day 4), the mice were kept in the experimental room in their home cage for 1 h, and then single-caged in the experimental cages only containing a clean cotton swab. On testing day (Day 5), the animals were moved to the experimental room and kept in their home cage for 1 h and then single-caged into experimental cages for 1 h without bedding containing an odorless cotton swab. After the habituation procedure, the mice were transferred into a new experimental cage for the habituation/dishabituation assay. Mice were tested for habituation and discrimination between five olfactory cues (Fig. 1b): odorless water (WAT), serving as a base line for general interest in the cotton swab, two artificial non-social odors: banana (BAN) and vanilla (VAN), and two social odors: opposite-sex urine (SOC-O) and same-sex urine (SOC-S).

*Habituation/dishabituation to non-social odors* For artificial non-social odors, we used banana and vanilla extract (McCormick & Company Inc., Hunt Valley, MD). The dominant compound in banana and vanilla odors are isoamyl acetate and vanillin, respectively. Both odor extracts were stored at room temperature and were freshly diluted 1:100 with distilled water for the experiment according to Yang & Crawley (2009) protocol^38^. Mice were exposed to a cotton swab soaked with 100 μL water or an odor solution for three 2-min trials (T1, T2 and T3) for a time course analysis. Each 6 min odor exposure was interrupted with 2 min inter-session (ISI) during which no odor was applied (Fig. 1b). New clean cotton swabs were used for each trial and each session.

*Habituation/dishabituation to social odors* For social odors, male and female mouse urine was used. Urine samples were collected as a mixture of fresh urine from 4–6 mice per gender in the morning before the onset of the behavioral assay^38^. Female urine sample was likely to be a mixture of urine from females at various stages of the estrous cycle^39,40^. Due to a possible effect of fluctuating levels of estrous cycle-dependent pheromones^41^ in the donor female urine mixture between experimental cohorts, behavioral experiments were counterbalanced between WT and *Fmr1* KO mice each day. Donor animals were healthy WT mice (3–4 month-old) from the same strain (FVB), who were not subjected to any experimental procedures. The urine samples were obtained by holding the donor mouse over the collection box. If needed, urination was induced by gentle abdominal pressure and any urine produced was collected with a pipette into the sterile tube. The obtained urine samples were diluted 1:5 with distilled water^42^ and stored at 4 °C. Each experimental group was first exposed to the urine obtained from opposite sex (SOC-O, males exposed to female urine and females exposed to male urine) and then the urine from same sex group (SOC-S, males exposed to male urine and females exposed to female urine) was presented. Procedures for the habituation/dishabituation to non-social odors task were repeated (Fig. 1b).

*Recording and analysis of olfactory behavior* Habituation/dishabituation tasks were conducted in a well-lit room. A JVC camcorder was placed in front of the animal cage, such that the recording showed the entirety of one side of the mouse cage. Following testing, the sniffing duration of the mouse was manually analyzed by an investigator who was blind to the genotype of the mouse. Sniffing duration was recorded from the point at which the mouse was oriented towards the cotton swab with its nose 0.5 cm from the tip. The investigator analyzed these videos on a 35.5 cm computer screen, therefore, the minimum distance between the mouse’s nose and the cotton swab tip appeared to be 1 cm. Habituation was analyzed by comparing the total sniffing duration between the first (T1) and last (T3) trial within one odor session. Dishabituation was analyzed by comparing the total sniffing duration in T3 of one odor session and T1 of the following odor session. In order to evaluate ability to discriminate, we calculated discrimination index (DI) by dividing the difference in sniffing duration time between the two odors (non-familiar odor [T1]—familiar odor [T3]) by the total amount of sniffing duration for both odors (non-familiar odor [T1] + familiar odor [T3]). DI was then multiplied by 100 to express it as a percentage^43^. DI equals to zero corresponds to a full preference towards non-familiar odor. Negative scores correspond to a preference towards familiar odor^43^. Male mice were euthanized after the experiments by using carbon dioxide followed by cervical dislocations. Female mice were additionally assessed for reproductive status and then euthanized after the experiments by using carbon dioxide followed by cervical dislocations.

*Assessment of female reproductive status* The stage of female estrous cycle was determined by cytological analysis of vaginal lavage immediately after the habituation/dishabituation test. The vagina was gently flushed with 10 μL of phosphate-buffered saline (PBS) using pipette with 10 μL tip and collected vaginal lavage was smeared on the slide. Next, vaginal smear containing cells were fixed using methanol for 10 min, stained with Wright-Giemsa Stain for 5 min following by rinsing in distilled water, and then coverslipped with Permount. The stage of estrous cycle was determined according to the absence, presence, or proportion of four basic cell types^44^. The females were next split into two groups based on the expected levels of gonadal and adrenal hormones: (1) proestrus and estrus with high or increasing hormone levels, and (2) metestrus and diestrus with low or decreasing hormone levels^45^.

### Histology

Male mice were anaesthetized by isoflurane and were transcardially perfused with PBS and 4% paraformaldehyde (PFA) dissolved in PBS. The brains were incubated for 24 h in 4% PFA for post-fixation and then cryoprotected using 30% sucrose dissolved in PBS for 48 h at 4 °C. After the cryoprotection, the brains were incubated in warm (37 °C) 11% gelatin dissolved 10% phosphate-buffered sucrose overnight, they were placed at 4 °C to harden. The gelatin blocks were stored in 30% sucrose dissolved in 4% PFA at 4 °C until next processing. The gelatin-embedded main olfactory bulb (OB) was cut in 40 μm coronal sections on cryostat (CM1860 UV; Leica Microsystems). The sections were collected in PBS, mounted on the glass slides, then left to dry overnight at room temperature. Dried brain sections were washed with xylene and rehydrated with descending gradient of ethanol (95% EtOH, 70% EtOH) and distilled water. Nissl bodies were stained in 0.2% cresyl violet for 15 min, rinsed in distilled water, dehydrated through an ascending gradient of ethanol (70% EtOH, 95% EtOH, and 100% EtOH), cleared by two changes of xylene, and then coverslipped with Permount.

### Stereological analysis of OB volume

Every third section of OB was imaged using Zeiss Axio Observer 7 microscope with 10 × objective and captured with camera Axiocam 503 (Carl Zeiss Microscopy GmbH). The images were then stitched with ZEN 2.3 software (Carl Zeiss Microscopy GmbH). The total volume of OB as well as the volumes of the glomerular layer (GL) and the granular cell layer (GCL) were estimated using Cavaleri principle and evaluated using ImageJ plugin Volumest^46^ with point-grid method counting^47^. The Gunderson coefficient of error (CE) for each animal quantified was always < 5%^48^. The mean CE is presented in the **Figure legend**. Data are reported as the total estimated volume (mm^3^) of OB, GL, or GCL or relative volume (%) of respective cell layer to the total OB volume. The total volume of the external piriform layer (EPL), mitral cell layer (MCL), and internal piriform layer (IPL), termed as EPL + MCL + IPL was calculated by subtracting GL and GCL volumes from the total OB volume. Sample size was determined by generally accepted from similar studies^49–51^.

### Immunostaining

Male mice were anaesthetized by isoflurane and were transcardially perfused with PBS and 4% PFA dissolved in PBS. The brains were incubated for 24 h in 4% PFA for post-fixation and then cryoprotected using 30% sucrose dissolved in PBS for 48 h at 4 °C. After the cryoprotection, the brains were embedded into the Tissue-Plus O.C.T. and stored in -80 °C for the next processing. The O.C.T.-embedded OB was cut in 20 μm coronal sections on cryostat (CM1860 UV; Leica Microsystems). The sections were collected directly on the glass slides and kept in −20 °C until the next immunostaining procedure. Samples were then treated in sodium citrate buffer for antigen retrieval for 15 min. After being washed twice with PBS for 5 min, the sections were incubated with blocking solution (5% goat serum, 0.01% Triton-X 100 in PBS) for 1 h and then incubated with the appropriate primary antibodies overnight at 4 °C. The following day, the sections were washed 4 times with PBS for 10 min, followed by incubation with secondary antibodies for 2 h at room temperature afterwards and then was washed 3 times in PBS for 5 min. DAPI was additionally stained for 20 min. After washing 3 times with PBS for 5 min and with 70% ethanol for 1 min, the sections were mounted with ProLong Gold Antifade Reagent. Immunostained sections were kept in 4 °C.

### Cell number quantification

Cell number quantification was performed in four defined regions of interest (ROIs) in the GL and GCL in anterior and posterior olfactory bulb. The ROIs were selected with respect to olfactory bulb morphology. Each ROI represents the olfactory bulb in its dorsal, ventral, medial, and lateral regions in the GL and GCL. The images were captured using Zeiss Axio Observer 7 microscope with a 40 × oil objective and Axiocam 503 mono camera (Carl Zeiss Microscopy GmbH) at a 10 µm range with 1 µm intervals, total of 11 sections. To analyze total number of the cells per ROI, DAPI^+^ cells were counted. NeuN^+^ neurons were quantified as the number of NeuN^+^ cells normalized by the number of DAPI^+^ cells. To analyze the subpopulation of periglomerular interneurons, CalB^+^, CalR^+^ and TH^+^ cells were counted and normalized by the number of DAPI^+^ cells. DAPI^+^ and NeuN^+^ cells were counted separately for the GL and GCL. CalB^+^, CalR^+^ and TH^+^ cells were counted only in the GL. The cell quantification was performed by ImageJ software (NIH) and the grand means were calculated for each animal and cellular marker. Each cell density value represents the average of 8 ROIs (4 ROIs from anterior and 4 ROIs from posterior OB part) per animal. Sample size (*n* = 4) was determined by generally accepted number from similar studies^52,53^.

### Statistical analysis

All data was analyzed using traditional statistical tests extended with a non-parametric permutational approach. We employed a permutational three-way ANOVA with repeated measurements to test whether genotype (between-group factor) or odor session and trial number (within-group factors) significantly affected total sniffing duration for non-social and social odors. The interactions of these factors were also evaluated. For two-sample, statistical model paired *t*-test (paired-sample design) or unpaired *t*-test (unpaired-sample design) were used. Analysis of covariance (ANCOVA) was used to analyze the differences between groups while statistically control the effect of an additional variable (covariate) on the dependent variable. ANOVA, ANCOVA and *t*-tests were performed with maximum of 5000 (ANOVA, ANCOVA) and 10,000 (*t*-tests) permutations. A Dirichlet regression was used to statistically assess the proportions of the GL, EPL + MCL + IPL, and GCL volumes (GL:EPL + MCL + IPL:GCL) in WT and *Fmr1* KO mice. All data are presented as mean ± SEM. The *n* represents biological replicates and can be found in the Figure Legends. Significance level was defined as *P* < 0.05. Statistical analyses were conducted using lmPerm and RVAideMemoire packages for R version 3.6.0.

## Results

### *Fmr1* KO males show an altered response during the olfactory habituation/dishabituation test

A modified olfactory habituation/dishabituation behavioral test^36^ was used to determine whether WT and *Fmr1* KO mice differ in their ability to distinguish between various non-social odors or social odors, as well as habituate to an odor after continuous exposure. We first evaluated the effect of the estrous cycle on female sniffing behavior during the olfactory habituation/dishabituation test by determining estrous and non-estrous statuses of both WT and *Fmr1* KO females. We did not find any significant differences in total sniffing duration between estrous and non-estrous females across all genotypes (*P* > 0.05; Supplementary Fig. 1). Thus, we pooled all females to evaluate subsequent analyses. Permutational three-way ANOVA test assessing whether genotype or odor session and trial number significantly affected total sniffing duration revealed no significant differences between genotypes for males (*F*_*(1,31)*_ = 0.36, *P* > 0.05; Table 1) or females (*F*_*(1,30)*_ = 2.97, *P* > 0.05; Table 1). This suggests that *Fmr1* KO mice show comparable olfactory habituation/dishabituation ability for odors as WT controls. However, in male mice, genotype interaction with within-group factors (i.e., session and trial) significantly affected total sniffing duration (Genotype*Session: *F*_*(4,124)*_ = 2.31, *P* < 0.05; Genotype*Trial: *F*_*(2,62)*_ = 2.85, *P* < 0.05; Genotype*Session*Trial: *F*_*(8,248)*_ = 1.68, *P* < 0.05; Table 1). These results indicate that our sample of *Fmr1* KO males have significantly altered olfactory responsiveness when exposed to various non-social and social odors. To determine which odor component contributes to this effect, the non-social and social odors were next analyzed separately.

**Table 1:** The ANOVA comparison of total sniffing duration in WT and *Fmr1* KO mice during the olfactory non-social and social habituation/dishabituation test.

	Males	Females
Genotype	*F*_(1,31)_ = 0.36, n.s.	*F*_(1,30)_ = 2.97, n.s.
Session	*F*_(4,124)_ = 33.29, *P* < 0.001	*F*_(4,120)_ = 9.05, *P* < 0.001
Trial	*F*_(2,62)_ = 168,39, *P* < 0.001	*F*_(2,60)_ = 115.16, *P* < 0.001
Genotype*Session	*F*_(4,124)_ = 2.31, *P* < 0.05	*F*_(4,120)_ = 2.03, n.s.
Genotype*Trial	*F*_(2,62)_ = 2.85, *P* < 0.05	*F*_(2,60)_ = 0.63, n.s.
Session*Trial	*F*_(8,248)_ = 66.30, *P* < 0.001	*F*_(8,240)_ = 10.74, *P* < 0.001
Genotype*Session*Trial	*F*_(8,248)_ = 1.68, *P* < 0.05	*F*_(8,240)_ = 0.73, n.s.

Statistical significance of between-group factor (Genotype) and within-group factors (Session and Trial) as well as their interactions are shown.

Permutational ANOVA with repeated measurements; n.s.: non significant

## *Fmr1* KO mice demonstrate altered olfactory responsiveness to non-social odor

**Fig. 2:**
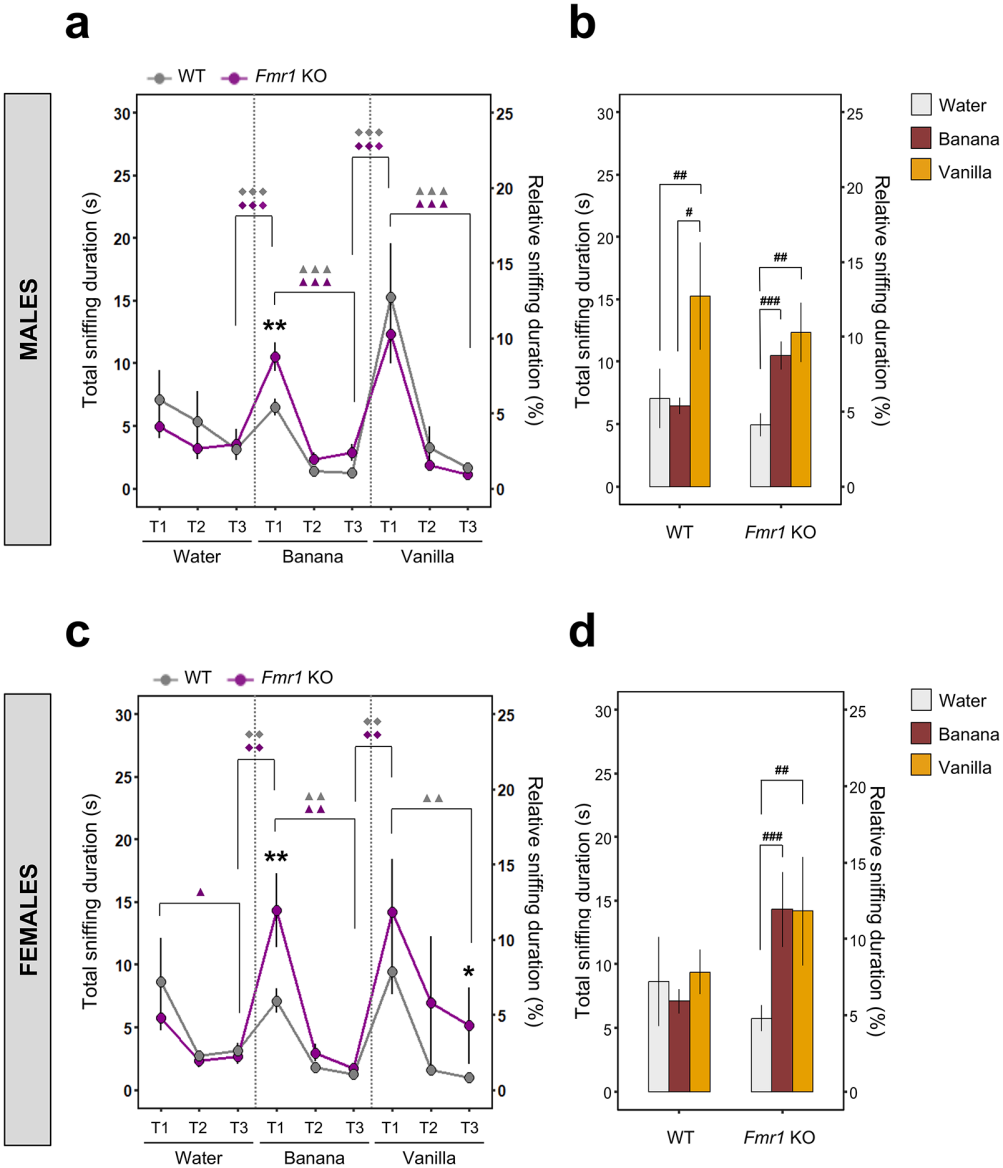
Spontaneous non-social olfactory habituation/dishabituation and discrimination task in WT and *Fmr1* KO mice. **(a)** Absolute (s) and relative (%) total sniffing duration during the non-social olfactory habituation/dishabituation test in WT and *Fmr1* KO males (WT: *n* = 16, *Fmr1* KO: *n* = 17). **(b)** Discrimination between two non-social odors (banana and vanilla) in WT and *Fmr1* KO males. **(c)** Absolute (s) and relative (%) total sniffing duration during the non-social olfactory habituation/dishabituation test in WT and *Fmr1* KO females (WT: *n* = 17, *Fmr1* KO: *n* = 15). **(d)** Discrimination between two non-social odors (banana and vanilla) in WT and *Fmr1* KO females. For the non-social olfactory habituation/dishabituation test **(a, c)** three odorant-paired sessions were used in following order: 1) water, 2) banana, and 3) vanilla. Each session consists of three consecutive trials (T1–T3) with 2 min inter-session interval. The statistical significances of the habituation (T1 vs. T3) and dishabituation (T3 vs. T1 of following session) for each genotype were evaluated using permutational paired *t*-test, $^{\blacktriangle}$*P* < 0.05, $^{\blacktriangle \blacktriangle}$*P* < 0.01, $^{\blacktriangle \blacktriangle \blacktriangle}$*P* < 0.001 for habituation, and ^◆◆^*P* < 0.01, ^◆◆◆^*P* < 0.001 for dishabituation. The symbol colors match with the appropriate genotype (grey for WT, magenta for *Fmr1* KO). The statistical differences between genotypes were calculated using permutational non-paired *t*-test, *P < 0.05, **P < 0.01. For non-social odor discrimination **(b, d)** the comparison between first trials (T1) of two distinct odors (banana and vanilla) was evaluated using permutational paired *t*-test, ^#^*P* < 0.05, ^##^*P* < 0.01, ^###^*P* < 0.001. All data represent means ± SEM.

The non-social olfactory habituation/dishabituation task consisted of three odor sessions: water (WAT, odorless control as a baseline for the mouse’s interest in the cotton tip), banana (BAN), and vanilla (VAN) (Fig. 1). Sniffing duration across all three trials of exposure to WAT (T1, T2, T3) showed no significant differences in both WT and *Fmr1* KO males (*P* > 0.05; Fig. 2a). However, males of both genotypes showed significant decline in sniffing duration from the first (T1) to the last trial (T3), exhibiting habituation when exposed to BAN and VAN (*P* < 0.001; marked with the triple triangle in Fig. 2a). Additionally, males of both genotypes also dishabituated when exposed to a new non-social odor (*P* < 0.001; marked with the triple diamond in Fig. 2a). A DI analysis revealed no significant difference between genotypes (*P* > 0.05; Supplementary Table 1). However, *Fmr1* KO males did exhibit a significantly longer sniffing duration during the first exposure to BAN (BAN T1) after WAT (WAT T3), compared to WT males (*P* < 0.01; marked with the double asterisk in Fig. 2a). Further analysis of total sniffing durations during T1 sessions for all three odors revealed that both WT and *Fmr1* KO males exhibited a significant increase in sniffing duration in VAN T1 compared to WAT T1 (*P* < 0.01; marked with the double hashtag in Fig. 2b). Interestingly, WT males showed an increased sniffing duration in VAN T1 compared to BAN T1 (*P* < 0.05; marked with the single hashtag in Fig. 2b), which was not observed in *Fmr1* KO males (*P* > 0.05; Fig. 2b). In contrast, *Fmr1* KO males displayed an increased sniffing duration in BAN T1 compared to WAT T1 (*P* < 0.001; marked with the triple hashtag in Fig. 2b), which was not seen in WT males (*P* > 0.05; Fig. 2b).

In females, similar differences between *Fmr1* KO and WT male mice were observed with some exceptions. These exceptions include the fact that WT females did not show significant habituation to WAT (*P* > 0.05; Fig. 2c), while *Fmr1* KO females showed significant habituation when exposed to WAT (T1 vs. T3, *P* < 0.05; marked with the single triangle in Fig. 2c). Additionally, WT females showed a gradual and significant decrease in VAN T1, T2, and T3 (*P* < 0.01; marked with the double triangle in Fig. 2c), and no significant habituation was observed in *Fmr1* KO females (*P* > 0.05; Fig. 2c). However, significant habituation was observed in response to BAN in both genotypes (*P* < 0.01; marked with the double triangle in Fig. 2c). Similar to males, both genotype females also dishabituated when exposed to a new non-social odor (*P* < 0.01; marked with the double diamond in Fig. 2c). Lastly, a DI analysis revealed increased dishabituation ability between WAT T3 and BAN T1 only in *Fmr1* KO females compared to WT females (*P* < 0.01; Supplementary Table 1). It is also worth noting that *Fmr1* KO females spent significantly longer sniffing duration during BAN T1 (*P* < 0.01; marked with the double asterisk in Fig. 2c) and VAN T3 (*P* < 0.05; marked with the single asterisk in Fig. 2c) compared to WT females. Similar to *Fmr1* KO males, *Fmr1* KO females showed significantly longer sniffing duration during BAN T1 (*P* < 0.001; marked with the triple hashtag in Fig. 2d) and VAN T1 (*P* < 0.01; marked with the double hashtag in Fig. 2d) compared to WAT T1, while no differences across odors were observed in WT females (*P* > 0.05; Fig. 2d).

Lastly, we conducted a permutation *t*-test in order to determine sex differences in the total sniffing duration in non-social olfactory habituation/dishabituation test. We found that *Fmr1* KO females demonstrated significantly longer sniffing duration during VAN T3 (F: 5.11 ± 3.07 s, M: 1.14 ± 0.37 s, *P* < 0.05; Supplementary Table 2) as compared with *Fmr1* KO males, while WT males and females did not differ in total sniffing duration across all non-social odors (*P* > 0.05; Supplementary Table 2).

### *Fmr1* KO males demonstrate decreased olfactory responsiveness to female urine

**Fig. 3:**
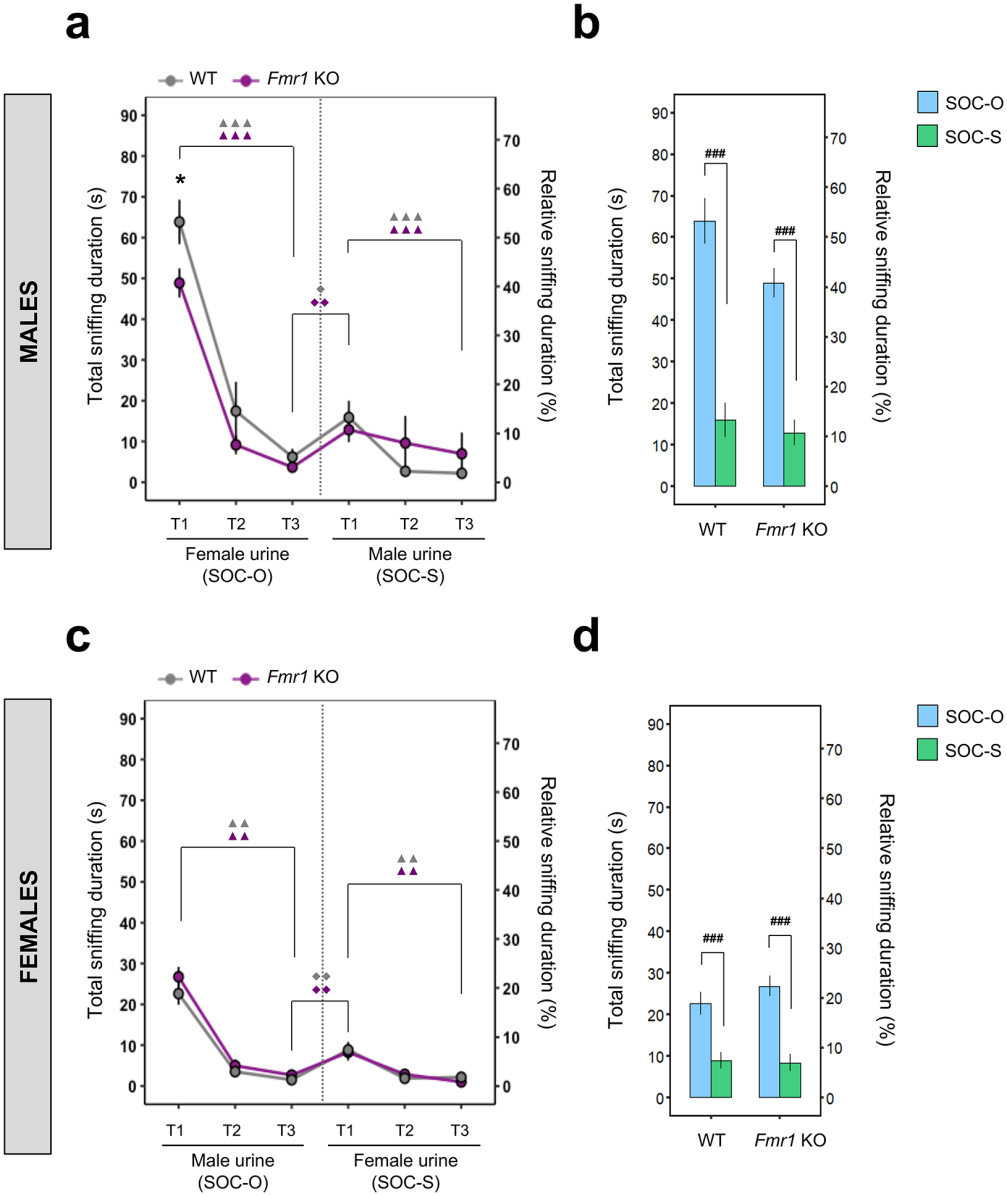
Spontaneous social olfactory habituation/dishabituation and discrimination task in WT and *Fmr1* KO mice. **(a)** Absolute (s) and relative (%) total sniffing duration during the social olfactory habituation/dishabituation test in WT and *Fmr1* KO males (WT: *n* = 16, *Fmr1* KO: *n* = 17). **(b)** Discrimination between two sex-group specific social odors (female and male urine, respectively) in WT and *Fmr1* KO males. **(c)** Absolute (s) and relative (%) total sniffing duration during the social olfactory habituation/dishabituation test in WT and *Fmr1* KO females (WT: *n* = 17, *Fmr1* KO: *n* = 15). **(d)** Discrimination between two sex-group specific social odors (male and female urine, respectively) in WT and *Fmr1* KO females. For the social olfactory habituation/dishabituation test **(a, c)** two sex-group specific odorants were used. The sex-group related odor-paired sessions were performed in following order: 1) opposite sex-group odor (SOC-O) and 2) same sex-group odor (SOC-S). Each session consists of three consecutive trials (T1–T3) with 2 min inter-session interval. The statistical significances of the habituation (T1 vs. T3) and dishabituation (T3 vs. T1 of following session) for each genotype were evaluated using permutational paired *t*-test, $^{\blacktriangle \blacktriangle}$*P* < 0.01, $^{\blacktriangle \blacktriangle \blacktriangle}$*P* < 0.001 for habituation, and ^◆^*P* < 0.05, ^◆◆^*P* < 0.01 for dishabituation. The symbol colors match with the appropriate genotype (grey for WT, magenta for *Fmr1* KO). The statistical differences between genotypes were calculated using permutational non-paired *t*-test, *P < 0.05. For social odor discrimination **(b, d)** the comparison between first trials (T1) of SOC-O and SOC-S was evaluated using permutational paired *t*-test, ^###^*P* < 0.001. All data represent means ± SEM.

WT and *Fmr1* KO males habituated when exposed to female urine (SOC-O, *P* < 0.001; marked with the triple triangle in Fig. 3a) as well as male urine (SOC-S, *P* < 0.001; marked with the triple triangle in Fig. 3a). Both male genotypes dishabituated when exposed to a new social odor (SOC-O T3 vs. SOC-S T1, WT: *P* < 0.05; marked with the single diamond, *Fmr1* KO: *P* < 0.01; marked with the double diamond in Fig. 3a). A DI analysis also revealed no significant difference between genotypes (*P* > 0.05; Supplementary Table 1). *Fmr1* KO males showed significantly shorter sniffing duration during the initial exposure to female urine odor (SOC-O T1), compared to WT males (*P* < 0.05; marked with the single asterisk in Fig. 3a). Furthermore, we found that both WT and *Fmr1* KO males had significantly longer sniffing duration when exposed to female urine odor (SOC-O), compared to male urine odor (SOC-O T1 vs. SOC-S T1, *P* < 0.001; marked with the triple hashtag in Fig. 3b).

In females, WT and *Fmr1* KO mice habituated to male and female urine (*P* < 0.01; marked with the double triangle in Fig. 3c). Both genotype females dishabituated when exposed to a new social odor (SOC-O T3 vs. SOC-S T1, *P* < 0.01; marked with the double diamond in Fig. 3c). A DI analysis revealed no significant differences between genotypes (*P* > 0.05; Supplementary Table 1). Additionally, WT and *Fmr1* KO females demonstrated significantly longer sniffing duration to male urine odor compared to female urine odor (SOC-O T1 vs. SOC-S T1,* P* < 0.001; marked with the triple hashtag in Fig. 3d).

Lastly, we performed a permutation *t*-test in order to determine sex differences in the total sniffing duration in social olfactory habituation/dishabituation test. We have found that WT males showed significantly longer sniffing duration in SOC-O across all trials (SOC-O T1; M: 63.8 ± 5.45 s, F: 24.0 ± 2.49 s; *P* < 0.01, SOC-O T2; M: 17.4 ± 7.22 s, F: 3.73 ± 0.66 s; *P* < 0.05, and SOC-O T3; M: 6.18 ± 2.05 s, F: 1.61 ± 0.24 s; *P* < 0.05; Supplementary Table 2) compared to WT females. *Fmr1* KO males demonstrated significantly longer sniffing duration during SOC-O T1 only, compared to *Fmr1* KO females (M: 48.9 ± 3.58 s, F: 26.8 ± 2.44 s; *P* < 0.01; Supplementary Table 2). Notably, both genotypes did not demonstrate any significant sex differences when exposed to SOC-S (*P* > 0.05; Supplementary Table 2).

### *Fmr1* KO males show increased volume of olfactory bulb

**Fig. 4:**
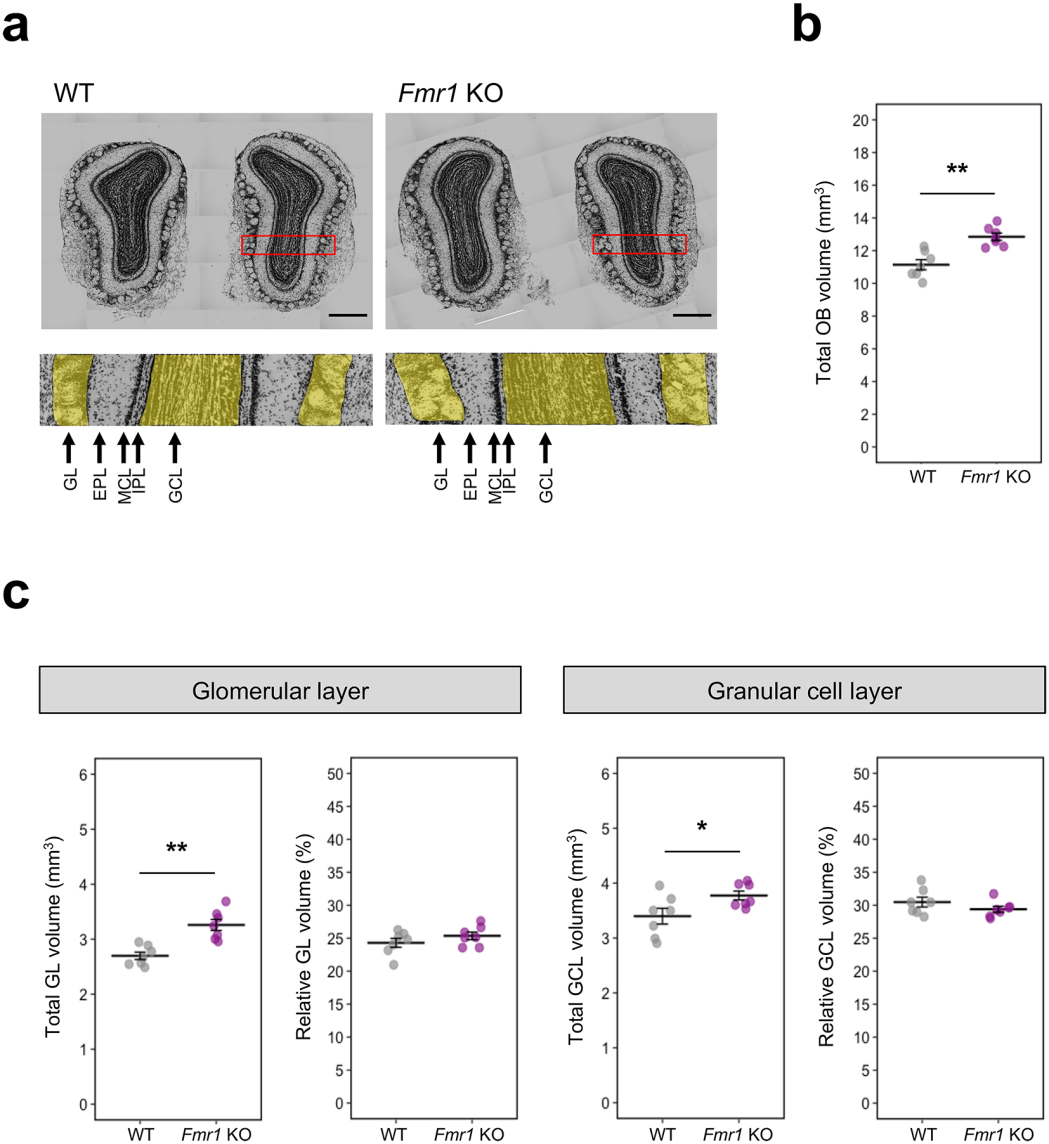
Stereological volume analysis of total olfactory bulb, glomerular layer and granular cell layer in WT and *Fmr1* KO mice. **(a)** The representative Nissl-stained coronal section of olfactory bulb (upper panels)with schematic detailed description of the layers (lower panels): GL, glomerular layer; EPL, external plexiform layer; MCL, mitral cell layer; IPL, internal plexiform layer; GCL, granule cell layer. Analyzed glomerular and granular cell layers are highlighted in yellow. **(b)** Stereological analysis of total olfactory bulb volume (WT: CE = 0.03 ± 0.008; *Fmr1* KO: CE = 0.03 ± 0.003). **(c)** Left panels: The volume analysis of total (left) and relative (right) glomerular layer (WT: CE = 0.02 ± 0.001; *Fmr1* KO: CE = 0.02 ± 0.001). Right panels: The volume analysis of total (left) and relative (right) granular cell layer (WT: CE = 0.04 ± 0.023; *Fmr1* KO: CE = 0.03 ± 0.002). Relative volumes are expressed as a percentage of total OB volume. All volume measurements were quantified from Nissl-stained sections. Volumes were estimated by the Cavalieri method, and the mean CE was calculated. Data were analyzed by two sample permutational *t*-test. All data represent means ± SEM. *P* values were calculated between WT (*n* = 7) and *Fmr1* KO (*n* = 7) mice, *P < 0.05, **P < 0.01.

Previous studies have shown that olfactory function can also correlate with the volume of olfactory bulb (OB)^26,27^. Thus, we performed a non-biased stereological analysis of the main OB to determine volume differences between *Fmr1* KO and WT male (Fig. 4a). Because we did not observe any significant effect of genotype between-group factors (or its interaction with other within-group factors) in females during the olfactory habituation/dishabituation test, we performed olfactory bulb volume analyses only in male mice. Our results revealed that *Fmr1* KO males had significantly higher OB volume compared to their WT male littermates (*P* < 0.01; marked with the double asterisk in Fig. 4b). We also found that the total OB volume significantly correlated with body weight in WT mice (Spearman’s rank correlation: *R* = 0.893, *P* < 0.05; Supplementary Fig. 2). Thus, we employed a permutation one-way analysis of covariance (ANCOVA) for OB volume using body weight as a covariate. This ANCOVA analysis revealed that the total OB volume was significantly higher in *Fmr1* KO mice than WT mice (*F*_(1,11)_ = 47.31, *P* < 0.001; Supplementary Fig. 2).

Moreover, *Fmr1* KO mice have significantly larger GL and GCL total volume compared to WT mice (GL: *P* < 0.01; marked with the double asterisk, GCL: *P* < 0.05; marked with the single asterisk in Fig. 4c). However, when we analyzed GL and GCL volume percentage in relation to the total OB volume, we did not find any significant difference between genotypes (*P* > 0.05; Fig. 4c). Building on these observations, we expanded our analysis to examine other layers of the olfactory bulb, including the external piriform layer (EPL), mitral cell layer (MCL), and internal piriform layer (IPL) by subtracting GL and GCL volumes from the total OB volume. Total volume of these three layers (EPL + MCL + IPL) also showed a significant increase in *Fmr1* KO mice compared to WT (*P* < 0.01; marked with double asterisk in Supplementary Fig. 3a). However, we did not observe any significant difference between *Fmr1* KO and WT mice in the combined volumes of EPL, MCL, and IPL (EPL + MCL + IPL) relative to the total OB volume (*P* > 0.05; Supplementary Fig. 3b). Lastly, the proportional representation of GL, EPL + MCL + IPL, and GCL volumes (GL:EPL + MCL + IPL:GCL) did not differ between genotypes (*P* > 0.05; Supplementary Fig. 3c). This suggests that the relative distribution of these specific layers is consistent across the two genotypes. Thus, our results demonstrate a general enlargement of olfactory bulb.

### No significant difference in OB cell population between WT and *Fmr1* KO males

**Fig. 5:**
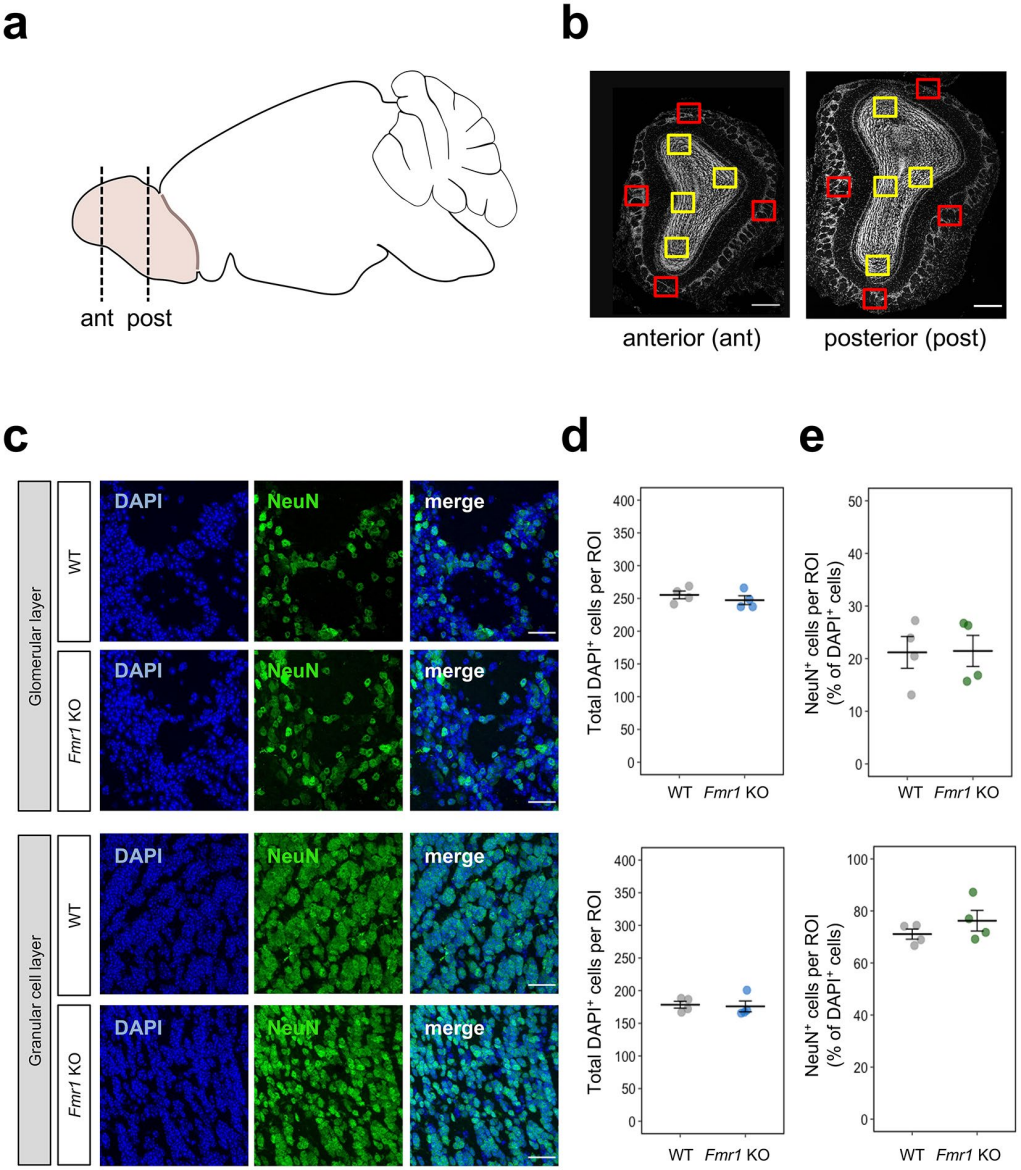
Cell analysis of the olfactory bulb in WT and *Fmr1* KO mice. **(a)** Scheme of brain sagittal view with the highlighted olfactory bulb. The dotted lines represent anterior (ant) and posterior (post) coronal sections where the cell quantification was evaluated. **(b)** Representative images of anterior and posterior coronal sections of olfactory bulb with highlighted regions of interest (ROIs) where the images were taken and cell analysis was performed. Red ROIs are for glomerular layer and yellow ROIs are for granular cell layer. Scale bar represents 300 μm. **(c)** Nuclear staining with DAPI^+^ (blue) and immunostaining of NeuN^+^ (green) cells in glomerular and granular cell layer. Scale bar represents 30 μm. **(d)** Quantification of total DAPI^+^ cells per ROI in glomerular and granular cell layer. **(e)** Quantification of NeuN^+^ cells among total DAPI^+^ cells (%) per ROI in glomerular and granular cell layer. The data for each animal were analyzed separately for glomerular and granular cell layer as an average of 8 images taken from ROIs in anterior and posterior part of olfactory bulb. Data were analyzed by two sample permutational *t*-test. All data represent means ± SEM. *n* = 4.

**Fig. 6:**
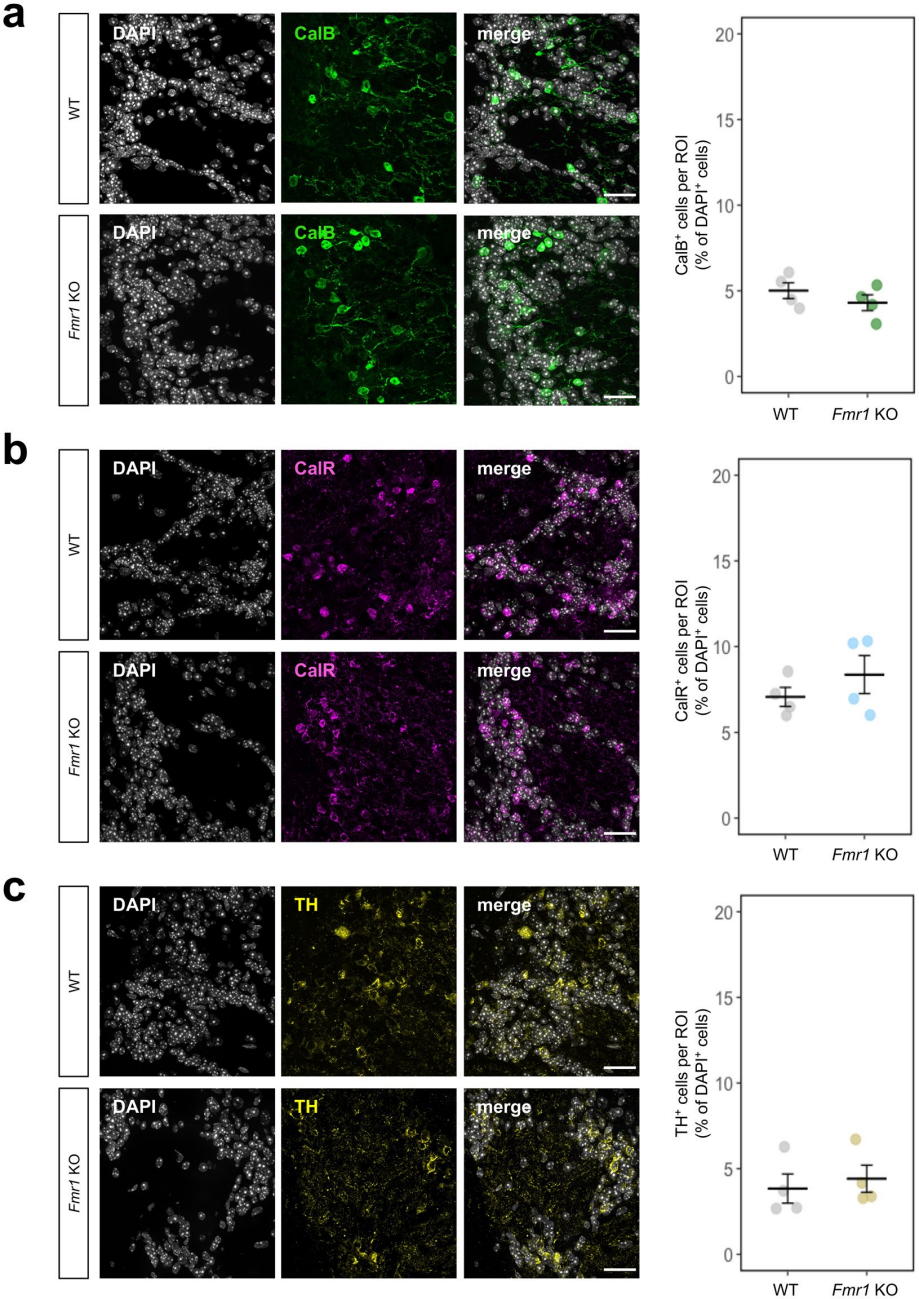
Cell analysis of glomerular interneurons in the olfactory bulb of WT and *Fmr1* KO mice. **(a)** Left: immunostaining of Calbindin-positive (CalB^+^) cells (green). Right: Quantification of CalB^+^ cells among total DAPI^+^ cells (%). **(b)** Left: immunostaining of Calretinin-positive (CalR^+^) cells (magenta). Right: Quantification of CalR^+^ cells among total DAPI^+^ cells (%). **(c)** Left: immunostaining of Tyrosine hydroxylase-positive (TH^+^) cells (yellow). Right: Quantification of TH^+^ cells among total DAPI^+^ cells (%). Scale bar represents 30 μm. The data for each animal were analyzed as an average of 8 images taken from ROIs in glomerular layer in anterior and posterior part of olfactory bulb. Data were analyzed by two sample permutational *t*-test. All data represent means ± SEM. *n* = 4.

After observing significant changes in the volume of the GL and GCL, we further evaluated the cell density in these layers from anterior and posterior regions of the OB (Figs. 5a and b). We found no significant difference between WT and *Fmr1* KO mice in cell density (DAPI^+^) in both GL (upper panels) and GCL (lower panels) (*P* > 0.05; Figs. 5c and d). Additionally, the proportion of neurons (NeuN^+^) among all DAPI^+^ cells were not different between WT and *Fmr1* KO mice in both cell layers (*P* > 0.05; Figs. 5c and e). One of the explanations for abnormal sensory processing in FXS could be the dysfunctional intrinsic excitability or impaired inhibition due to a loss of inhibitory neurons^54^. Thus, we further quantified the density of modulatory interneurons. Our results revealed that the density of CalB^+^, CalR^+^, and TH^+^ cells is not significantly different in *Fmr1* KO mice compared to WT (*P* > 0.05; Figs. 6a–c). We also did not see any significant differences between genotypes for the density of CalB^+^, CalR^+^, and TH^+^ cells in the dorsal, ventral, medial, and lateral regions of the GL (*P* > 0.05; Supplementary Fig. 4).

## Discussion

In this study, we used an olfactory habituation/dishabituation test to analyze olfactory behavior in *Fmr1* KO mice. We demonstrated that *Fmr1* KO male mice exhibit aberrant olfactory social response to the female urine. We also found the olfactory bulb in *Fmr1* KO male mice have significantly increased OB volume compared to their WT littermates. The ubiquitous expression of FMRP in the olfactory system^55^ suggests that its deficiency may influence odorant sensing, as well as higher bulbo-cortical processing, such as odor discrimination, social motivation, and cognitive functions. Given that *Fmr1* KO mice showed olfactory habituation/dishabituation patterns similar to WT, these behaviors may be a simple, non-associative form of learning that is not significantly altered in *Fmr1* KO mice.

*Fmr1* KO mice exhibited increased sniffing duration during their first-time exposure to the BAN odor (BAN T1) compared to WT mice. This elevated sniffing duration observed in *Fmr1* KO mice during BAN T1 could be driven by multiple possibilities, which include potential consequences due to a decreased olfactory sensitivity and a deficit in olfactory-driven aversive behavior. Notably, previous studies have reported increased olfactory investigation as a sign of diminished sensitivity in *Fmr1* KO mice^25^. Since we observe this behavior only when mice were exposed to BAN odor for the first time, but not to VAN odor, the manifestation of olfactory sensory deficit in *Fmr1* KO mice could be also dependent on odorant-specific sensitivity threshold, learning (habituation) processes, and motivation to explore the cotton swab. Therefore, another possible explanation would be that rodents demonstrate a high sensitivity to isoamyl acetate (the dominant compound of BAN odor), with detection thresholds as low as 3.6 ppb^56^ which can potentially lead to aversive behavior. In contrast, vanillin (the dominant compound of VAN odor) tends to evoke neutral or positive responses, likely due to its role in enhancing appetite^57^. The lack of significant difference in sniffing duration for the VAN odor suggests that the olfactory abnormality observed in *Fmr1* KO mice may be more specific to aversive stimuli, rather than a general olfactory deficit. This could be attributed to amygdala function, which plays a key role in processing aversive odors^58^. Thus, differences in sniffing behaviors in response to BAN and VAN odors could be explained by possible disruptions in neural circuits involved in fear and aversion in *Fmr1* KO mice. These multiple possible scenarios can be investigated in future studies.

In contrast to increased *Fmr1* KO mice activity in sniffing exploration of newly presented odorant, *Fmr1* KO males showed significantly reduced interest to explore female urine odor. Mouse urine consists of a plethora of distinct volatile compounds that vary according to the sex, strain and social status of individual mice^59–61^. It is hypothesized that urine scent is a natural olfactory component that promotes social communication in rodents, and thus can serve as a measure of social motivation^62^. Sociosexual motivation in rodent males is driven by dopamine release in the medial preoptic area^63^. Dopaminergic modulation in the olfactory bulb serves to optimize its sensitivity to changes in the chemosensory environment^64,65^. Interestingly, dysfunctional dopamine signaling has been reported in several studies in *Fmr1* KO mice^66–69^. However, we did not find any differences in the density of neurons stained against tyrosine hydroxylase (TH), an enzyme that catalyzes the formation of a dopamine precursor in glomerular layer of olfactory bulb, between *Fmr1* KO and WT mice.

Given the reported relationship between the olfactory bulb volume and olfactory function^26,27^, we hypothesize that observed functional abnormalities and reported structural changes in cell connectivity^32,70^ in *Fmr1* KO mice can also be reflected by the neuroanatomical properties of their olfactory bulb. Although previous studies have shown significant volume changes in various brain regions of *Fmr1* KO mice^71,72^ the olfactory bulb volume was reported to be intact^71^. In contrast to these studies, which employed magnetic resonance imaging (MRI) in order to analyze brain neuroanatomical characteristics in *Fmr1* KO mice, we have used an unbiased stereological approach to analyze the main olfactory bulb as well as its layers. We have shown that olfactory bulb in *Fmr1* KO mice is enlarged compared to WT controls, which recapitulates the findings in rat models of FXS^73^. Based on our analyses of the GL, GCL, and the combined volumes of the EPL, MCL, and IPL (EPL + MCL + IPL), which demonstrate no significant difference in their relative volume of each layer in relation to the total volume of the olfactory bulb between *Fmr1* KO and WT males, we can conclude that the olfactory bulb is uniformly enlarged in *Fmr1* KO males. Since we did not find any differences in density of selected cell-type specific populations between WT and *Fmr1* KO mice in glomerular as well as granular cell layer, this suggests that the enlarged olfactory bub volume could be caused by multiple potential factors such as abnormal structural cell connectivity or cell population abnormalities. Notably, cholecystokinin-positive (CCK^+^) tufted cells refine odor representation by modulating mitral/tufted cells’ firing, which is crucial for accurate odor perception^74,75^. Their activity impacts odor detection/discrimination and they are important for behaviors related to social interaction, particularly those involving pheromones^75^. Disrupting CCK^+^ tufted cells impair the processing of social odors, thus affecting normal social behavior^75^. Thus, a future study could analyze CCK^+^ tufted cells of the OB from *Fmr1* KO and WT mice, which would be informative to elucidate a mechanism of abnormal olfactory-associated social behaviors observed in *Fmr1* KO mice^76^.

Atypical sensory processing, specifically olfactory, is seen in ASD and FXS patients^16,77^. In the theory of predictive coding^78,79^ it is hypothesized that sensory and social processes in autism are tightly connected^80,81^. Thye et al. (2018) proposed multiple mechanisms through which early sensory dysregulation in ASD could cascade into social deficits across development^82^. Brang & Ramachandran (2010) also reported that olfactory bulb dysgenesis results in reduced vasopressin and oxytocin receptor binding, which are related to social bonding^83^. However, the multisensory abnormalities in ASD suggest that the top-down expectation abnormalities could be attributed to a disproportionate reliance allocated to prior anticipation than the bottom-up abnormal processing^80^. Olfaction is relatively less studied in ASD and FXS as compared with other sensory modalities^77,82^, despite its relevant for eating behaviors, which are shown to be disrupted in ASD and FXS^84–86^. Therefore, it is worth it for future studies to pay closer attention to the roles of olfaction and gustation in these disorders.

In summary, findings from this study suggest that the lack of FMRP may lead to altered olfactory behaviors as well as a volume increase of the olfactory bulb. Future studies can further identify underlying mechanisms of these olfactory phenotypes observed in *Fmr1* KO mice.

## Supplementary Information

Supplementary Information 1.

## Data Availability

This study did not generate new unique reagents. Further information and requests for resources, analysis and methodology should be directed to and will be fulfilled by the corresponding author.

## Code availability

All data reported in this paper will be shared by the corresponding author upon request. This paper does not report original code. Any additional information required to reanalyze the data reported in this paper is available from the corresponding author upon request.
