# Supplementary material for: Altered olfactory responses in Fmr1 KO mice
Source: Sci Rep. 2025 Jan 23;15:2952. doi: 10.1038/s41598-024-80000-5 (PMC11758012; doi:10.1038/s41598-024-80000-5)
Supplement: Supplementary file 1 — Supplementary Information 1. [file 41598_2024_80000_MOESM1_ESM.pdf]

Supplementary Material

Altered olfactory responses in *Fmr1* KO mice

Jan Tuma[1,2], Amtul-Noor Rana[1], Teena Philip[1], Jeong Ben Park[1] and Hye Young Lee[1]*

[1]The Department of Cellular and Integrative Physiology, The University of Texas Health Science Center at San Antonio, San Antonio, TX, USA

[2]Department of Pathophysiology, Faculty of Medicine in Pilsen, Charles University, Alej Svobody 1655/76, 323 00 Plzen, Czech Republic

* Correspondence to: Hye Young Lee

Email to: leeh6@uthscsa.edu

TABLE OF CONTENTS

Supplementary Figure 1

Supplementary Table 1

.

Supplementary Table 2

Supplementary Figure 2

Supplementary Figure 3

Supplementary Figure 4

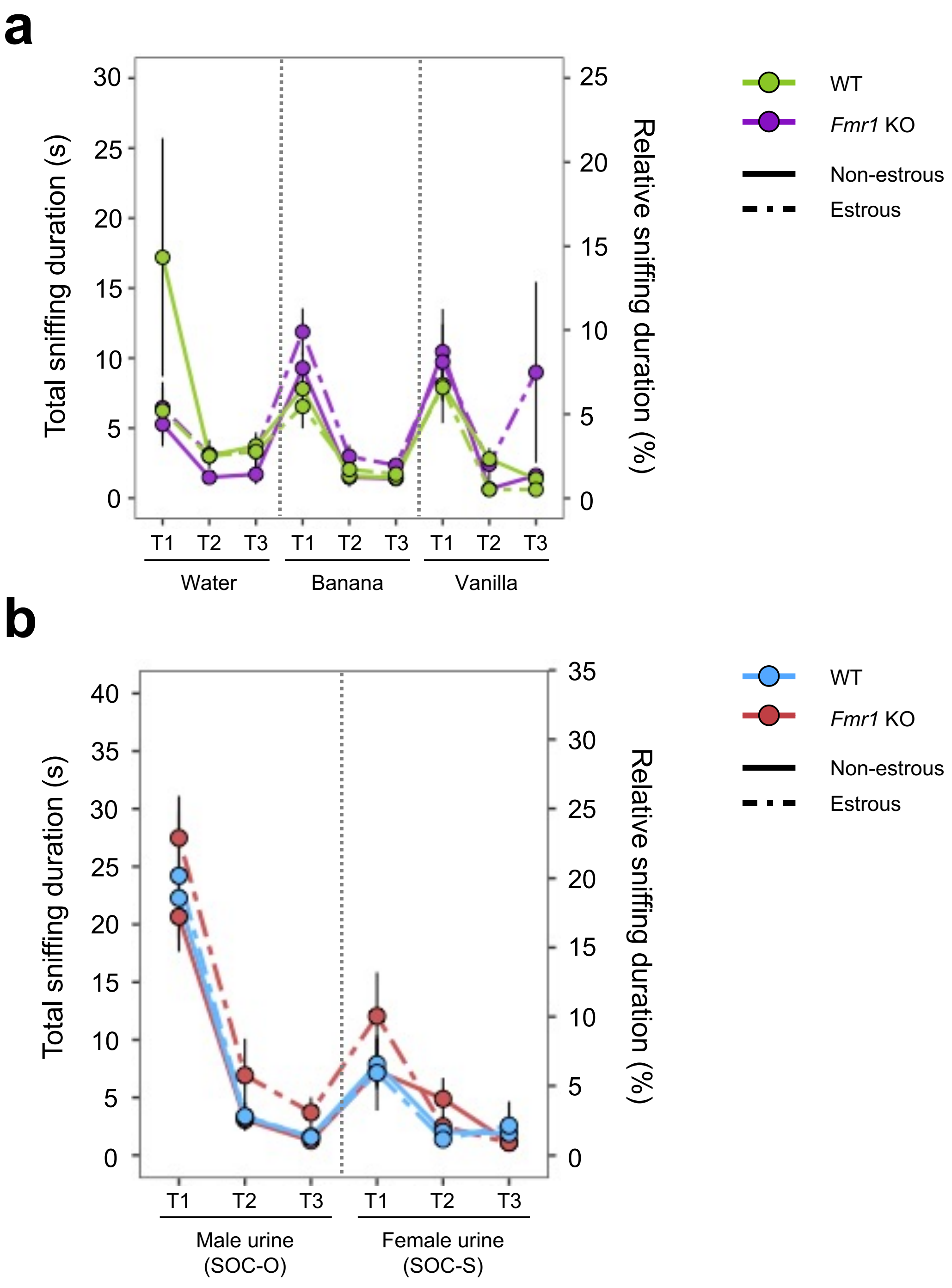

Supplementary Figure 1. The effect of female reproductive status on spontaneous olfactory habituation/dishabituation task in WT and *Fmr1* KO females. (a) Absolute (s) and relative (%) total sniffing duration during the non-social olfactory habituation/dishabituation test in WT and *Fmr1* KO females. **(b)** Absolute (s) and relative (%) total sniffing duration during the social olfactory habituation/dishabituation test in WT and *Fmr1* KO females. Non-estrous group: WT: *n* = 9, *Fmr1* KO: *n* = 8; estrous group: WT: *n* = 8, *Fmr1* KO: *n* = 7. All data represent means ± SEM.

Supplementary Table 1. Differences in the discrimination between familiar and new odor in WT and *Fmr1* KO mice.

	WT	*Fmr1* KO	*t*	*P*
**Males**				
WAT T3/BAN T1 DI	37.81	55.29	1.32	n.s.
BAN T3/VAN T1 DI	76.33	60.62	-1.51	n.s.
VAN T3/SOC-O T1 DI	94.19	95.08	0.41	n.s.
SOC-O T3/SOC-S T1 DI	46.53	54.63	0.56	n.s.
**Females**				
	WT	*Fmr1* KO	*t*	*P*
WAT T3/BAN T1 DI	30.02	69.04	3.37	< 0.01
BAN T3/VAN T1 DI	59.75	62.91	0.27	n.s.
VAN T3/SOC-O T1 DI	85.45	75.56	-1.11	n.s.
SOC-O T3/SOC-S T1 DI	45.43	52.81	0.55	n.s.

DI: Discrimination index

$$DI = \frac{(T1 - T3)}{(T1 + T3)} \times 100$$

WAT: Water
BAN: Banana
VAN: Vanilla
SOC-O: opposite sex-group odor
SOC-S: same sex-group odor
T1: Trial 1 (non-familiar odor)
T3: Trial 3 (familiar odor)
Permutational *t*-test
n.s.: non significant

Supplementary Table 2. Sex differences in total sniffing duration during the non-social and social habituation/dishabituation task in WT and *Fmr1* KO mice.

**Non-social odor**						
	**Trial 1**		**Trial 2**		**Trial 3**	
**WT**	*t*	*P*	*t*	*P*	*t*	*P*
WAT	0.49	n.s.	-1.03	n.s.	0.22	n.s.
BAN	0.93	n.s.	0.96	n.s.	0.1	n.s.
VAN	-1.14	n.s.	-0.88	n.s.	-1.41	n.s.
Fmr1 KO	*t*	*P*	*t*	*P*	*t*	*P*
WAT	0.59	n.s.	-0.8	n.s.	-0.59	n.s.
BAN	1.25	n.s.	0.71	n.s.	-1.48	n.s.
VAN	0.39	n.s.	1.02	n.s.	1.37	0.044
**Social odor**						
	**Trial 1**		**Trial 2**		**Trial 3**	
**WT**	*t*	*P*	*t*	*P*	*t*	*P*
SOC-O	-6.64	0.002	-1.89	0.014	-2.22	0.018
SOC-S	-1.41	n.s.	-0.96	n.s.	0.07	n.s.
Fmr1 KO	*t*	*P*	*t*	*P*	*t*	*P*
SOC-O	-4.96	0.002	-1.44	n.s.	-0.69	n.s.
SOC-S	-1.22	n.s.	-0.94	n.s.	-1.08	n.s.

WAT: Water
BAN: Banana
VAN: Vanilla
SOC-O: opposite sex-group odor
SOC-S: same sex-group odor
Permutational *t*-test
n.s.: non significant

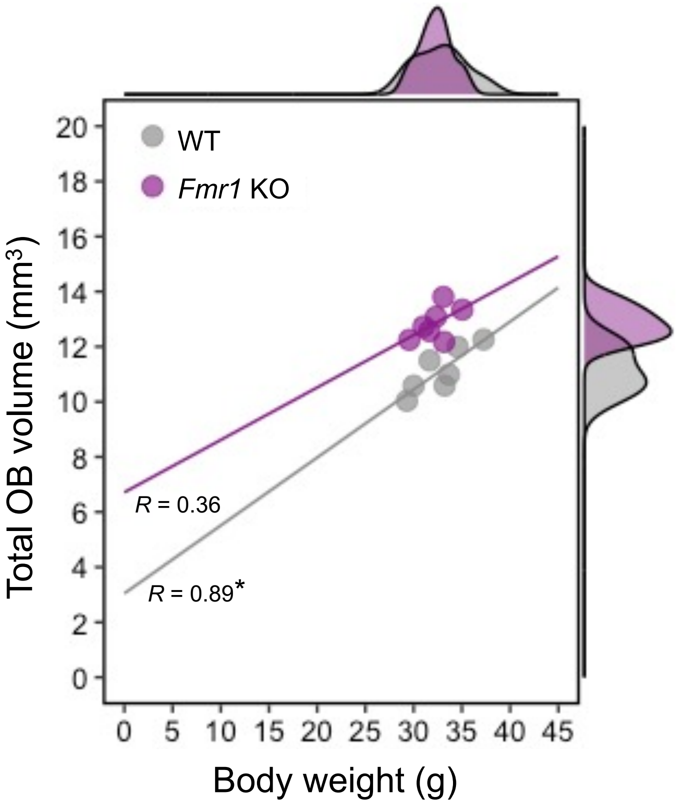

Supplementary Figure 2. Relation between olfactory bulb volume and body weight in WT and *Fmr1* KO males. Regression lines yielded by permutational one-way ANCOVA ($F_{(1,11)}$ = 47.31, P < 0.001). R represents Spearman correlation (*P < 0.05).

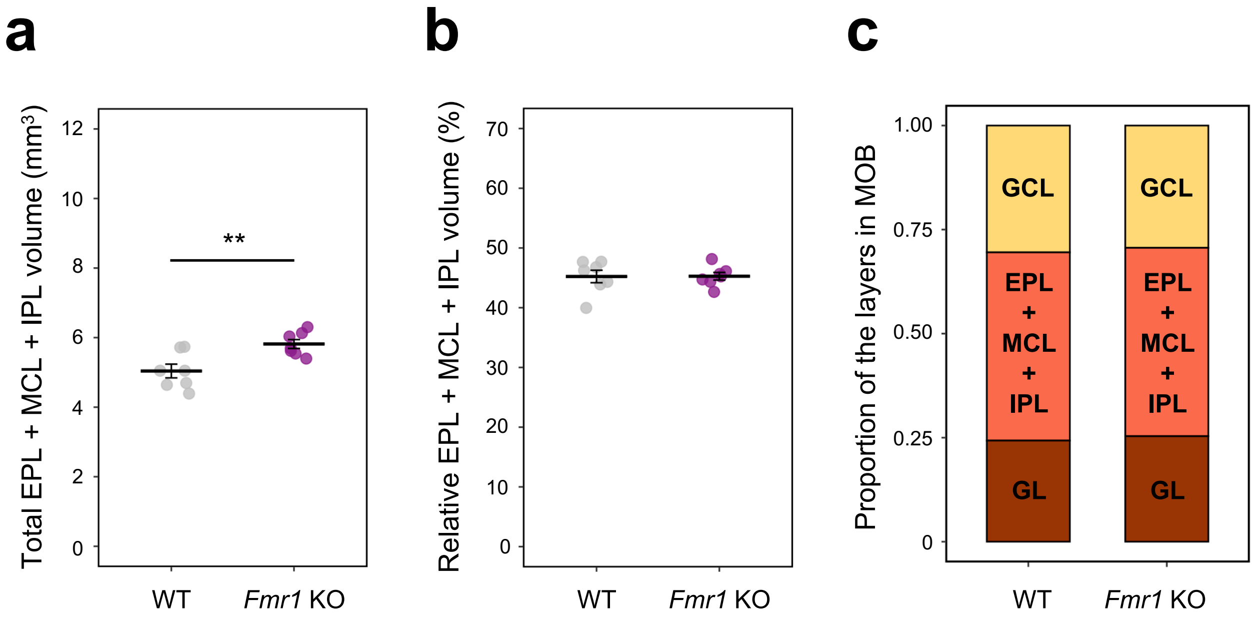

Supplementary Figure 3. Stereological volume analysis of the EPL, MCL, and IPL calculated by subtracting GL and GCL volumes from the total OB volume. (a) Total volume of EPL, MCL and IPL (EPL + MCL + IPL). **(b)** Relative volume of EPL, MCL, and IPL (EPL + MCL + IPL) expressed as a percentage of total OB. Data were analyzed by two sample permutational *t*-test. Data represent means ± SEM. *P* values were calculated between WT (n = 7) and *Fmr1* KO (n = 7) mice, **$P < 0.01$. **(c)** Average ratios of distinct cellular layers within the olfactory bulb for WT (n = 7) and *Fmr1* KO (n = 7) mice. Each bar shows the mean of proportions of GL, EPL + MCL + IPL, and GCL volumes (GL:EPL + MCL + IPL:GCL), calculated as averages across individual mice.

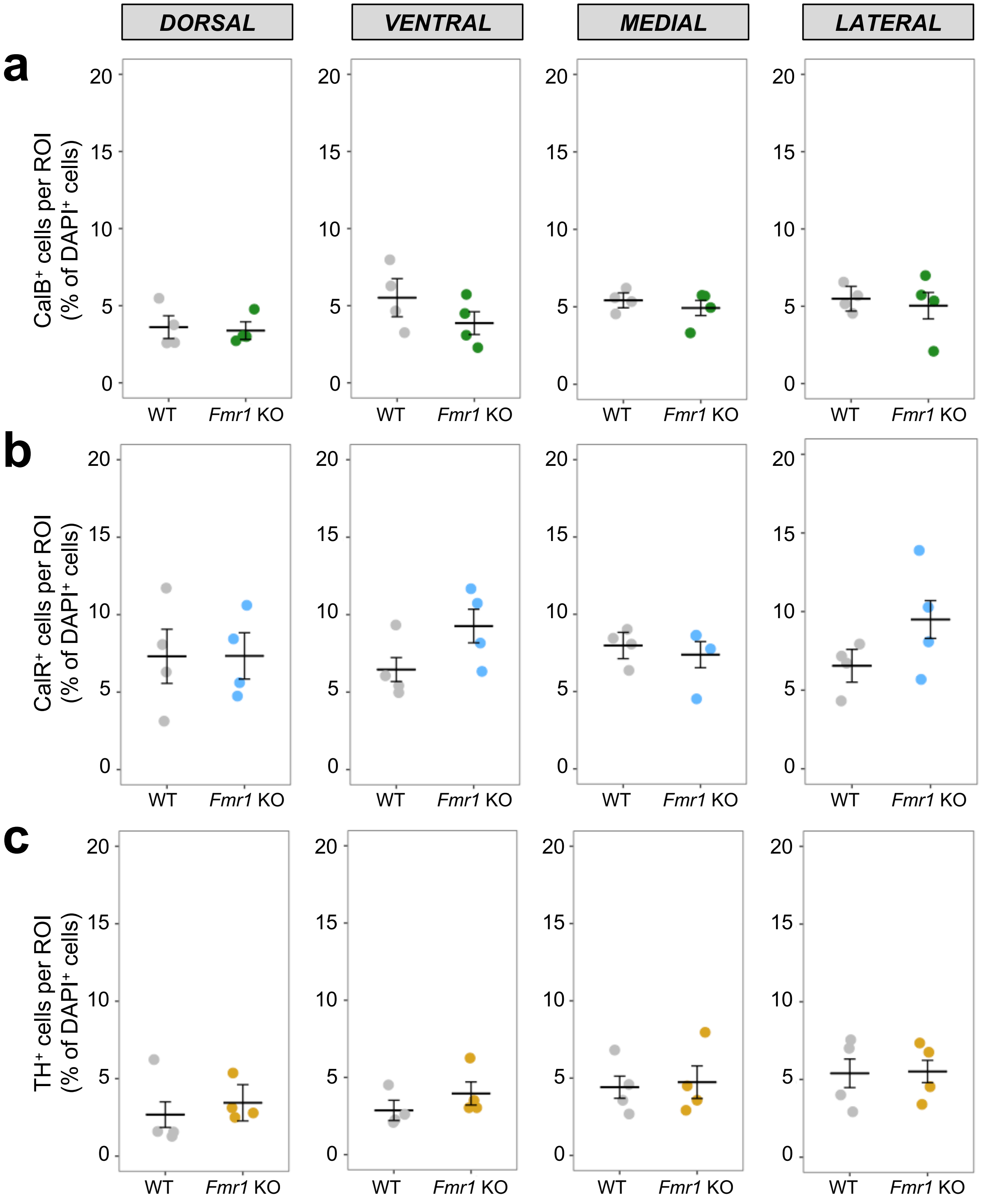

Supplementary Figure 4. Cell analysis of glomerular interneurons in the olfactory bulb from WT and *Fmr1* KO mice in the dorsal, ventral, medial, and lateral regions. (a) Quantification of $CalB^+$ cells among total $DAPI^+$ cells (%). **(b)** Quantification of $CalR^+$ cells among total $DAPI^+$ cells (%). **(c)** Quantification of TH^+ cells among total $DAPI^+$ cells (%). The data for each animal were analyzed and presented as an average of 2 images taken from glomerular layer in anterior and posterior part of olfactory bulb. All data represent means ± SEM. n = 4.
